# Association of Circulating T Follicular Helper Cells With Idiopathic Optic Neuritis and Neuromyelitis Optica Spectrum Disorders

**DOI:** 10.3389/fneur.2021.638473

**Published:** 2021-09-22

**Authors:** Qian Wu, Binbin Yang, Jiawei Wang

**Affiliations:** Department of Neurology, Tong Ren Hospital of Capital Medical University, Beijing, China

**Keywords:** idiopathic optic neuritis, NMOSD, Tfh cells, AQP4-Ab, CD4^+^CXCR5^+^PD-1, CD4^+^CXCR5^+^ T cells

## Abstract

**Background:** T follicular helper cells (Tfh cells) play an important role in activating B lymphocytes and may associate with idiopathic Optic Neuritis (ON) and Neuromyelitis Optica Spectrum Disorders (NMOSD).

**Objective:** This study aimed to examine the potential role of Tfh cells in pathogenesis of idiopathic ON and NMOSD.

**Methods:** Circulating CD4^+^CXCR5^+^ and CD4^+^CXCR5^+^PD-1^+^ cells in 46 idiopathic ON and 68 NMOSD patients as well as 28 healthy controls were examined by flow cytometry before treatment. Serum AQP4 antibody, Expended Disability Status Scale (EDSS) and Visual Outcome Scale (VOS) were detected before and after treatment.

**Results:** The percentages of circulating CD4^+^CXCR5^+^ and CD4^+^CXCR5^+^PD-1^+^Tfh cells in CD4^+^ cells (%) were significantly increased in idiopathic ON and NMOSD compared with those of healthy controls (*p* < 0.01). No significant difference of Tfh cells in blood and cerebral spinal fluid (CSF) was found between ON and NMOSD patients. The percentages of CSF, CD4^+^, CXCR5^+^, and CD4^+^CXCR5^+^PD-1^+^ cells in CD4^+^ cells (%) were positively correlated with those of the blood (*r* = 0.5781, *r* = 0.6079, *p* = 0.0076, and *p* = 0.0045, respectively). EDSS scores of NMOSD group were higher than those of ON group and the time course of NMOSD patients was longer than that of ON patients (*p* < 0.01). After methylprednisolone treatment, both EDSS and VOS scores were significantly decreased at discharge compared with before treatment (*p* < 0.01). There was no significant correlation among Tfh cell percentages in CD4^+^ cells, CSF leukocytes, CSF protein, annual recurrence rate, EDSS and VOS scores between two groups (*p* > 0.05).

**Conclusion:** The Circulating T follicular helper cells were increased in both idiopathic ON and NMOSD.

## Introduction

Idiopathic optic neuritis (ON) is a demyelinating inflammation of the optic nerve caused by autoimmune reactions targeting the optic nerve, which typically affects young adults ranging from 18 to 45 years of age. Patients usually present with acute reduction of visual acuity, orbital pain exacerbated by eye movements, dyschromatopsia, and afferent pupillary defect with or without optic disc edema.

Neuromyelitis optica spectrum disorder (NMOSD) is a rare and severe autoimmune disease of the central nervous system (CNS) driven by pathogenic antibodies mainly directed against aquaporin-4 (AQP4-Ab), which targets the AQP4 water channels of astrocytes in CNS and preferentially involves the optic nerve, spinal cord and posterior medullary area. NMOSD affects all races and ages, however it is more popular in Asian population and has a striking female preponderance. Because of its severely disabling relapses, NMOSD has a much higher early morbidity and mortality than multiple sclerosis (MS) (1). AQP4 antibody was detected in the serum of about 70% of NMOSD patients, suggesting a B cell-mediated immune pathology. However, there are still some NMOSD patients with negative AQP4 antibody, in which different immune mechanism might exist.

T follicular helper cell (Tfh cell) is a group of recently found subset of CD4^+^. T cells, which plays an important role in activating B lymphocytes. The stimulated B cells then produce antibodies that induce humeral immune responses. Tfh cells typically express CXC chemokine receptor 5 (CXCR5), inducible co-stimulator (ICOS), programmed death 1 (PD-1), CD40 ligand (CD40L) and other surface molecules. These characteristics are obviously different from other T helper cell subgroups. Studies have shown that CD4^+^CXCR5^+^ cell percentage in CD4^+^T cells is elevated in the blood of patients with autoimmune diseases such as Systemic Lupus Erythematosus (SLE) and Myasthenia Gravis (MG) and is associated with antibody production. Based on previous studies, we chose CD4^+^ CXCR5^+^ and CD4^+^ CXCR5^+^ PD-1^+^ T cells to represent Tfh cells in peripheral blood in this study (2–5).

Although Tfh cells are related to antibody-mediated immune response, it is unclear whether Tfh cells are involved in the pathogenesis of idiopathic ON and NMOSD. So far, changes of Tfh cells in idiopathic ON and comparative studies of Tfh cells between idiopathic ON and NMOSD have not been reported. Because idiopathic ON patients share some common courses of early NMOSD, such as frequent relapses and steroid sensitive, we postulated that its pathogenesis may be immune-mediated and Tfh cells might be involved in idiopathic ON and NMOSD. The purpose of the study was to investigate the potential role of Tfh cells in pathogenesis of idiopathic ON and NMOSD.

## Methods and Materials

### Patients and Clinical Information

This study was approved by the research ethics committee of Beijing TongRen Hospital. All participants signed informed consent. Acute phase idiopathic ON (*n* = 46) and high AQP4 level (AQP4 > 10 U/ml, *n* = 41) NMOSD patients who were hospitalized in our department from March 2015 to October 2017 were recruited. Idiopathic ON diagnosis and inclusion criteria: acute onset of unilateral or bilateral optic neuritis; no other CNS lesions, negative serum AQP4 antibody, do not meet the diagnosis of NMOSD or MS with optic neuropathy, and exclude other infectious factors. NMOSD diagnosis: NMOSD diagnostic criteria by the international optic neuromyelitis diagnostic unit (IPND) in 2015 were adopted (6). A total of 28 healthy volunteers were recruited as a control group (Table 1).

**Table 1:** Demographic and clinical characteristics in idiopathic ON, NMOSD, and HC groups.

**Clinical characteristic**	**Idiopathic ON**	**NMOSD**	**HC**	***p*-value**
*n*	46	41	28	
Male (%)	21 (45.7)	5 (12.2)	11 (39.3)	
Female (%)	25 (54.3)	36 (87.8)	17 (60.7)	*p* = 0.0002
Age, median (IQR)	34 (27–50)	37 (27–53)	29 (25–47)	*p* = 0.211
First attack (%)	34 (73.9)	10 (24.4)		*p* = 0.000
Relapses (%)	12 (26.1)	31 (75.6)		
Course (months), median (IQR)	0.75 (0.32–5.00)	24.0 (1.33–48.00)		*p* = 0.000
ARR, median (IQR)	1.63 (0.85–2.25)	1.00 (0.65–1.65)		*p* = 0.464
Time between the last attack to blood/CSF sampling (days), median (IQR)	9.43 (7.25–11.83)	8.83 (7.08–10.90)		*p* = 0.592
AQP4 value (IQR, U/ml)	1.14 (0.62–1.58)	58.24 (34.73–8.58)		*p* < 0.0001

*ON, optic neuritis; NMOSD, neuromyelitis optica spectrum disease; HC, health control; IQR, interquartile range; ARR, annual recurrence rate*.

All patients underwent MRI examination of brain and spinal cord. The history and clinical manifestation were recorded in detail.

Functional evaluation: Expanded Disability Status Scale (EDSS) scores were recorded before and after treatment. As vision impairment is the main complaint in both of ON and NMOSD patients of this study, Visual Outcome Scale (VOS) scores were also recorded.

### Sample Collection and Detection

Detection of AQP4 antibody: patients in the acute period who were not given corticosteroid or immunosuppressive treatment at least 1 month before admission had 2 ml of blood drawn prior to methylprednisolone treatment. The blood samples were added to EDTA anticoagulant tubes and regular tubes, respectively. Because our laboratory had no CBA method qualification certification, the ELISA method was adopted for AQP4 antibody test, using second-generation AQP4 ELISA Kit (ELISA RSR, AQP4 Ab Version 2) (7), which targeted the M23 peptide of AQP4-IgG. All procedures were done in strict accordance with the instructions. Results were detected by microplate reader (Thermo Fisher Scientific) and set ≥3 U/ml as the cut-off value for positive AQP4. To avoid false positive results, we excluded low AQP4 level (3–10 U/ml) NMOSD patients in the study and double confirmed with CBA method within our laboratory.

Detection of Tfh cells: Tfh cell percentages in CD4^+^ T cells were detected by flow cytometry. Samples were added with different fluorescence labeled anti-CD4-Ab anti-CXCR5-Ab, anti-PD-1 Ab, and the same type of control, then added 100 ul of fresh anticoagulant peripheral blood in each tube for 20 min of incubation without light. The cell lysate was added for 10 min to isolate lymphocytes (centrifugation, 1,500/min for 5 min). All tubes were then washed with PBS twice and analyzed by flow cytometry (BD FACSCalibur). Cerebral spinal fluid was taken from 20 patients, and Tfh cells in CSF were also detected by flow cytometry.

### Statistical Analysis

All results were statistically analyzed by SPSS 19.0 and Graphpad Prism 8.3.0. Categorical variables were described by counts and percentages, while non-normal distributed continuous and ordinal variables by median and interquartile ranges (IQRs). Demographic features of participants were compared using the Fisher exact-test or the Wilcoxon-test. Paired *t*-tests were used for comparing of EDSS and VOS scores before and after methylprednisolone treatment in NMOSD and ON groups. Non-parametric-tests with the Steel-Dwass multiple comparison were used to compare Tfh cells of the three groups. A Spearman rank correlation test was used for the correlation analysis between Tfh cells, AQP4 antibody and clinical parameters.

## Results

### Clinical Characteristics of the Idiopathic ON and NMOSD Patients

Relapses happened in 26% (12/46) of idiopathic ON and 75.6% (31/41) of NMOSD patients, with ARR being 1.63 and 1.00, respectively. The course of recurrent NMOSD was markedly longer than that of idiopathic ON. AQP4 values of ON group and NMOSD group (IQR) were 1.14 (0.62–1.58) U/ml and 58.24 (34.73, 78.58) U/ml, respectively (*p* < 0.0001).

### Comparison of Blood Tfh Cells and CSF Tfh Cell Between Idiopathic ON and NMOSD

**Figure 1:**
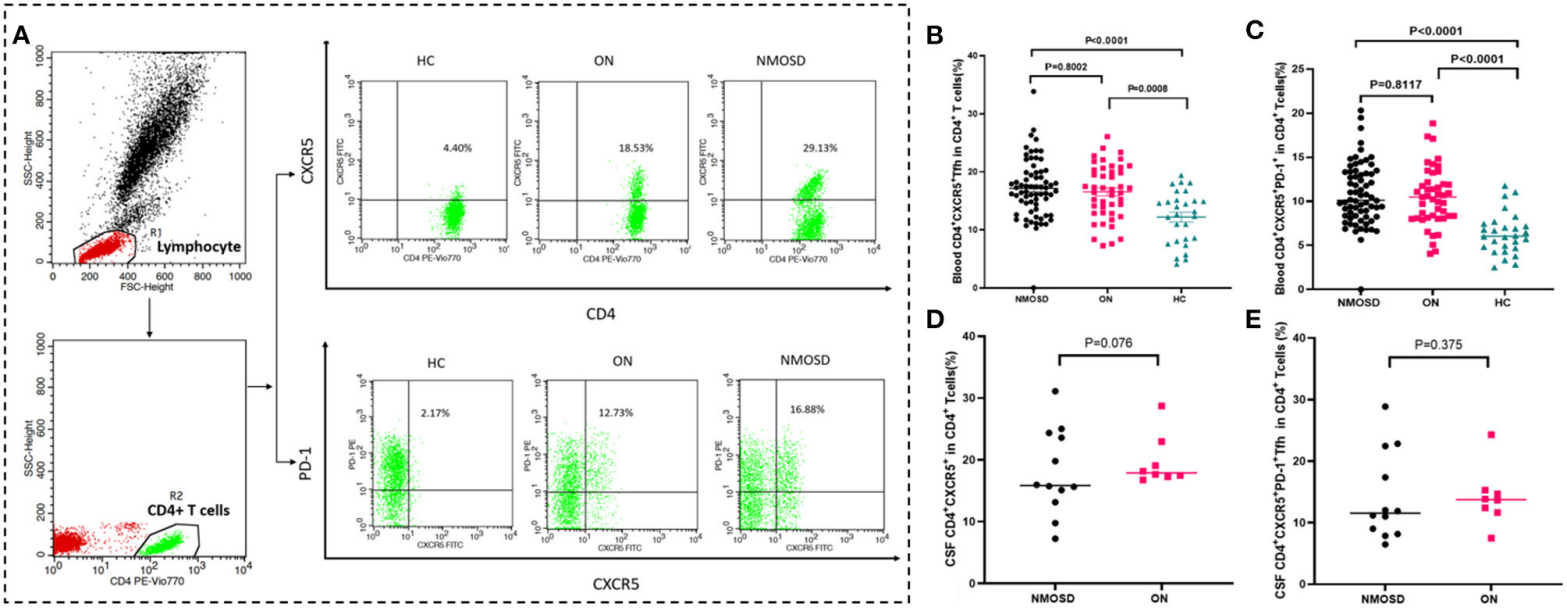
Comparison of Tfh cell percentage in CD4^+^ cells between HC, ON, and NMOSD groups. **(A)** The strategy for gating CD4^+^CXCR5^+^ and CD4^+^CXCR5^+^PD-1^+^ cells from CD4^+^ cells. CD4^+^ cells were first gated from the peripheral blood lymphocyte. The CD4^+^CXCR5^+^ and CD4^+^CXCR5^+^PD-1 cells were then gated from the CD4^+^ population. The data above the outlined area were the percentages of Tfh cells in CD4^+^cells in HC /ON/NMOSD groups. **(B,C)** Comparison of percentages of blood CD4^+^CXCR5^+^ and CD4^+^CXCR5^+^PD-1 cells in CD4^+^ T cells between NMOSD, ON, and HC groups. **(D,E)** Comparison of percentages of CSF CD4^+^CXCR5^+^ and CD4^+^CXCR5^+^PD-1 cells in CD4^+^ T cells between NMOSD, ON and HC groups.

The results indicated that the percentages of circulating CD4^+^CXCR5^+^ and CD4^+^CXCR5^+^PD-1 Tfh cells in CD4^+^ T cells were significantly higher in both ON group and NMOSD group than those of normal control group (*p* < 0.01; Figures 1A–C). There was no significant difference of circulating and CSF Tfh cell percentages CD4^+^ T cells between ON group and NMOSD groups (*p* > 0.05; Table 2 and Figures 1D,E).

**Table 2:** Changes of circulating follicular helper T cell percentages in idiopathic ON and NMOSD group.

**Group**	**Number**	**CD4^+^CXCR5^+^ (%)**	**CD4^+^CXCR5^+^-PD-1 (%)**
		**Median (IQR)**	**Median (IQR)**
NMOSD	41	16.22 (13.14–19.89)^**^	10.09 (8.89–13.09)^**^
ON	46	16.97 (12.96–20.33)^**^	10.49 (8.14–11.95)^**^
HC	28	12.92 (8.35–15.04)	6.05 (4.78–7.23)

*ON, optic neuritis; NMOSD, neuromyelitis optica spectrum disease; HC, health control; IQR, interquartile range*.

** *Represent p < 0.01 compared with normal control group. The values in the table represent the percentages of CXCR5^+^ and CXCR5^+^-PD-1^+^ cells in CD4^+^ T cells*.

**Figure 2:**
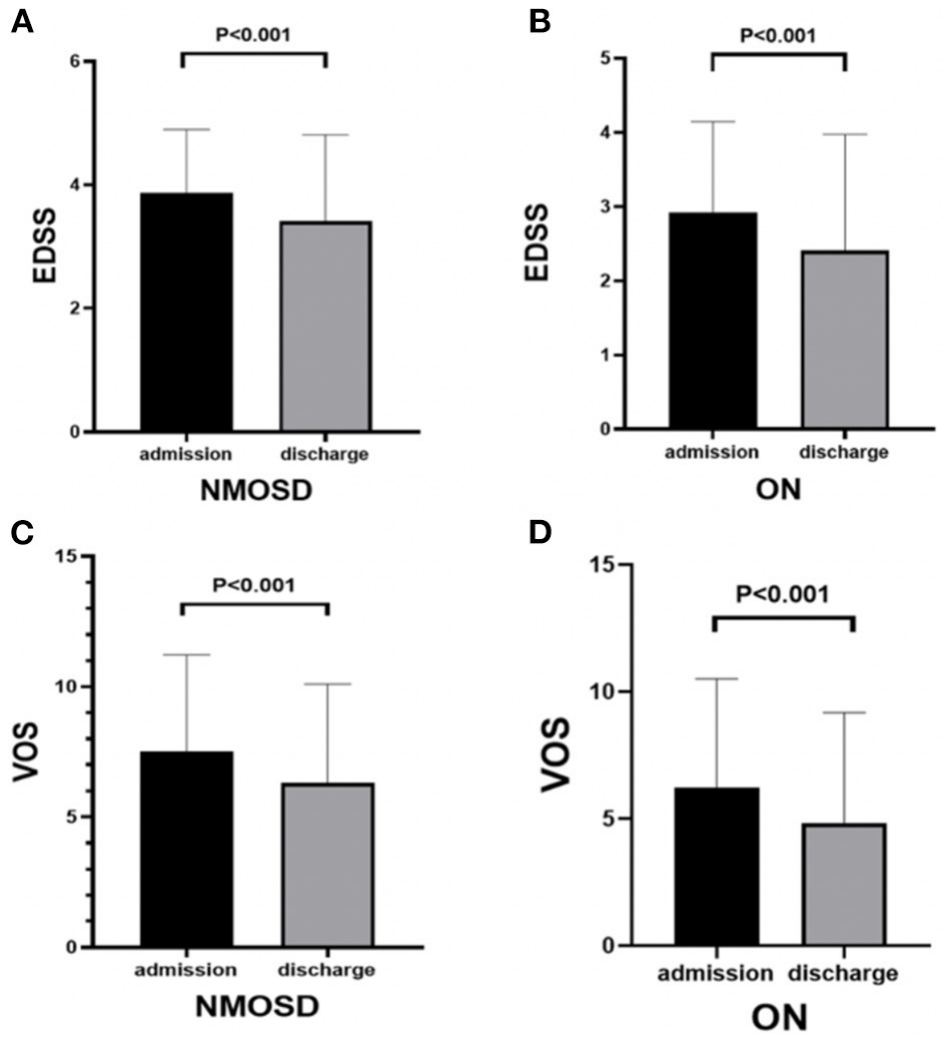
Comparison of EDSS and VOS scores before and after methylprednisolone treatment in NMOSD and ON groups: **(A)** EDSS scores of NMOSD at admission and discharge. **(B)** EDSS scores of ON at admission and discharge. **(C)** VOS scores of NMOSD at admission and discharge. **(D)** VOS scores of ON at admission and discharge.

The results showed that the scores of EDSS and VOS were significantly decreased at discharge after methylprednisolone treatment compared with those at admission in both groups (*p* < 0.01; Figures 2A–D).

**Figure 3:**
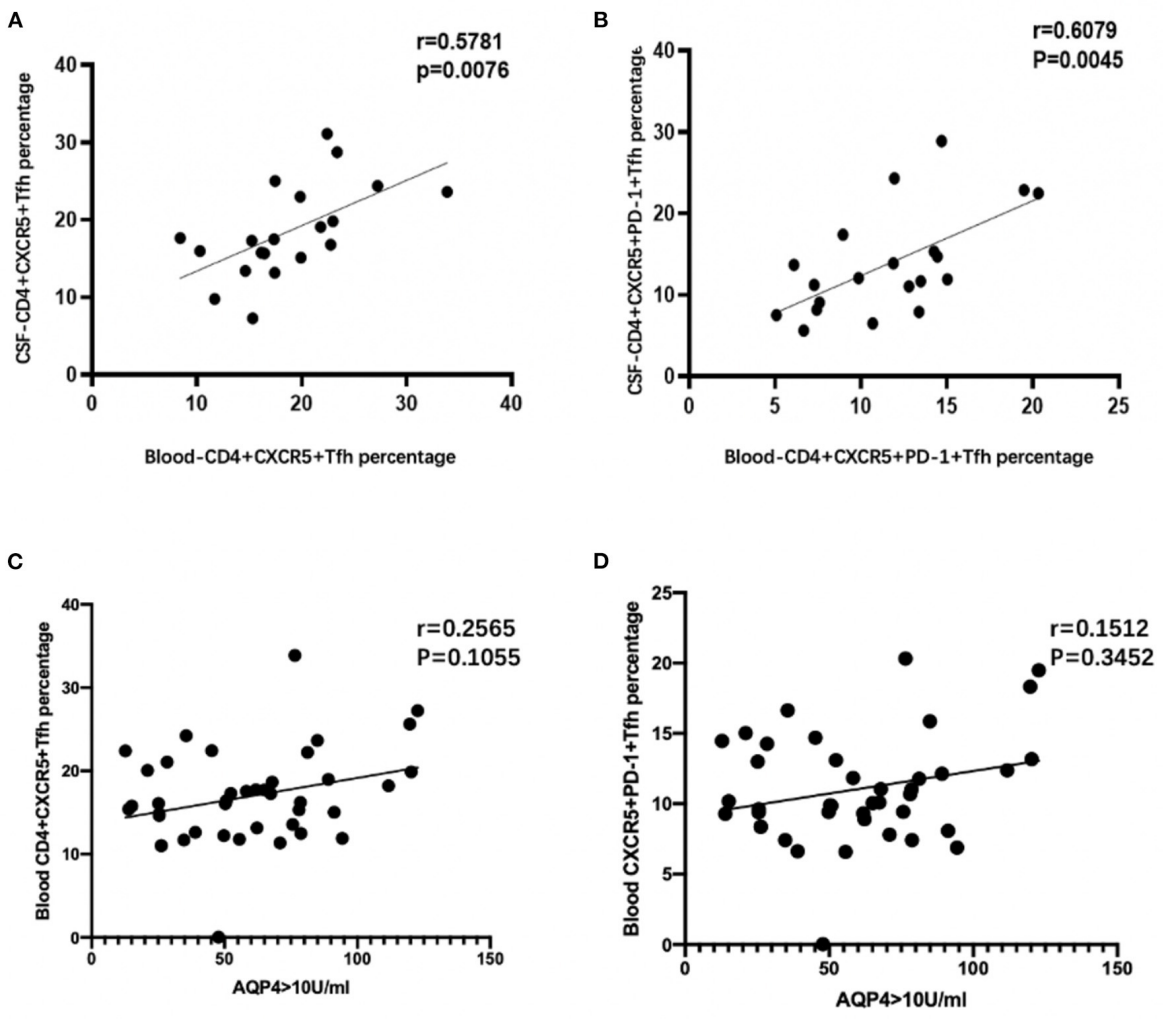
**(A,B)** Correlation analysis between CSF and blood Tfh cell percentages in CD4^+^ T cells of all patients with both blood and CSF samples. **(C,D)** Correlation analysis between blood Tfh cell percentages in CD4^+^ T cells and AQP4 value of NMOSD patients.

Correlation analysis showed that peripheral blood CD4^+^CXCR5^+^ Tfh cell percentages in CD4^+^ T cells were positively correlated with CD4^+^CXCR5^+^PD-1 (*r* = 0.835, *p* < 0.01), as well as in CSF (*r* = 0.881, *p* < 0.01). Cerebral spinal fluid and peripheral blood CD4^+^CXCR5^+^ and CD4^+^CXCR5^+^PD-1 showed a positive correlation (*r* = 0.5781, *r* = 0.6079, *p* = 0.00076, *p* = 0.00045; Figures 3A,B). No significant correlations were found between blood Tfh cell percentages and AQP4 values (*p* > 0.05; Figures 3C,D). There was no obvious correlation among Tfh, CSF leukocytes, CSF protein, annual recurrence rate, EDSS and VOS scores between two groups (*p* > 0.05).

## Discussion

In our study NMOSD patients showed obvious female predominance (31:10), while the male-female ratio in idiopathic ON group was almost the same (25:21). Results showed that the blood Tfh cell percentages of the two disease groups were significantly higher than in the normal control group. The percentages of Tfh cells in CD4^+^ T cells showed a positive correlation between blood and CSF. All the idiopathic ON patients' serum AQP4 antibody were negative, but the average annual recurrence rate of recurrent ON patients was not less than that of NMOSD patients. The blood Tfh cells of idiopathic ON were also significantly increased, and they were effective to steroids treatment, indicating the possible immune-mediated pathogenesis of idiopathic ON. We are not sure that whether the first-onset idiopathic ON patients of this study are expecting recurrences or will have developed NMOSD and MS in the future, but it is obvious that Tfh cells were involved in the pathogenesis of both diseases.

Up to now, a few studies have indicated that peripheral blood Tfh cells were increased in patients with NMOSD (8–12). Studies have shown that the percentages of peripheral blood CD4^+^CXCR5^+^PD-1^+^Tfh cells in NMOSD patients were increased compared with those in MS and healthy controls. Memory Tfh cells and interleukin-21 were increased in peripheral blood of patients with NMOSD, suggesting that Tfh is related to NMOSD.

Previous studies have indicated that the percentages of CCR7^−^ and CCR7^−^ICOS^+^ memory Tfh cells were positively correlated with the average ARR (9). However, our study revealed no significant correlation between CD4^+^CXCR5^+^ and CD4^+^CXCR5^+^PD-1^+^ Tfh cells with average ARR (*r* = 0.09, *p* > 0.05; *r* = 0.13, *p* > 0.05, respectively). The different results may be related to the different subtypes of Tfh cells studied. Tfh cells yield a population of cells that exit the GC and lymphoid tissues and then return to the circulation as a population of quiescent memory-type CD4^+^CXCR5^+^ T cells (13). Tfh cells are heterogeneous as there are different subsets of Tfh cells that have been studied, including CD4^+^CXCR5^+^ ICOS^+^, CD4^+^CXCR5^+^PD-1^+^, CD4^+^CXCR5^+^ ICOS^+^ PD-1^+^, CD4^+^CXCR5^+^ CD57^+^, and CD4^+^CXCR5^+^ IL-21^+^T cells (14). Since different groups used different markers for Tfh cell identification, further investigation is warranted to determine whether these markers are defining a different population of Tfh cells and whether different subsets of Tfh cells are correlated to ARR, states of disease, and prognosis. Our study further confirmed that Tfh cell percentages on CD4^+^ T cells were elevated in both the blood and CSF of NMOSD patients, and it was the first study to demonstrate that the Tfh cells were increased in the blood and CSF of idiopathic ON. We also found a significant positive correlation between blood and CSF Tfh cells. Therefore, we have deduced that activated blood Tfh cells may activate B lymphocytes to produce antibodies, which might enter the CNS through the blood-brain barrier and play a potential role in the pathogenesis of idiopathic ON and NMOSD.

In this study, EDSS and VOS scores of NMOSD and ON groups were significantly decreased at discharge after methylprednisolone treatment as compared with those at admission, indicating that methylprednisolone therapy is effective both for idiopathic ON and NMOSD in the acute stage. Feng et al. reported that corticosteroids might inhibit aberrant circulating Tfh cell proportions in patients with systemic lupus erythematosus (15). Nicolas et al. reported that the balance in Tfh cell subsets is altered in NMOSD patients and restored by rituximab (16, 17). Therefore, we speculated that the effects of corticosteroids for idiopathic ON and NMOSD in the acute stage might relate to inhibit aberrant circulating Tfh cell proportions and circulating Tfh cell proportions might be a potential indicator for immunosuppressive therapy in idiopathic ON and NMOSD in the acute stage.

The present study had some limitations. First, due to the rarity of idiopathic ON and NMOSD, the number of patients included in this study was limited. Studies of CSF samples in the healthy control and non-inflammatory disease control group need to be included. Second, CXCR5, ICOS, PD-1, CD40L and other surface molecules are all characteristically expressed by Tfh cells and are important for differentiate Tfh from other T helper cell subsets. Various markers of Tfh cells, as well as Tfh cell subsets are expected to be added to further clarify their involvement in the disease pathogenesis. Fourth, cell-based for the AQP4-IgG assessment (CBA) kit was not registered by CFDA and our laboratory had no CBA method qualification certification; therefore, we used the second-generation of AQP4 ELISA kit (RSR British Ltd, version 2) coated with the human recombinant M23 isoform of AQP4 for the study. Compared with the first generation of enzyme immunoassay kit (AQP4 M1 protein), the kit has higher sensitivity and specificity. International registration studies showed that the sensitivity of the second-generation ELISA kit was 77–89% (NMOSD) and the specificity was 95–99% (healthy people) (7, 18). At present, AQP4 ELISA is also commonly used to study NMOSD internationally, such as researches led by Mayo clinic and John Hopkins medical center (7, 9, 19, 20). However, several studies have found that ELISA method for AQP4 detection sometimes cause false positive results. To avoid false positive, we choose high titer AQP4 NMOSD patients (AQP4 > 10 U/ml) and confirmed with CBA method within our laboratory. Fifth, Although the blood Tfh cells were increased in both of idiopathic ON and NMOSD, they were not correlated to ARR, EDSS and VOS scores. Therefore, further prospective longitudinal studies with a greater number of patients and various Tfh cell markers are needed to confirm the role of Tfh cells in idiopathic ON and NMOSD.

## Conclusion

The circulating Tfh cells were increased in both idiopathic ON and NMOSD. But whether and how they were involved in the pathogenesis of the two diseases still needs further investigation.

## Data Availability Statement

The original contributions presented in the study are included in the article/supplementary material, further inquiries can be directed to the corresponding author.

## Ethics Statement

The studies involving human participants were reviewed and approved by Beijing TongRen Hospital, Capital Medical University. Written informed consent for participation was not required for this study in accordance with the national legislation and the institutional requirements.

## Author Contributions

QW contributed to conception and design, collection of blood and CSF samples, and interpretation of data, statistical analysis, and drafting and revision of the manuscript. BY contributed to detect the AQP4 antibody and Tfh cells. JW contributed to conception and was the study supervisor. All authors contributed to the article and approved the submitted version.

## Funding

This study was funded by Beijing TongRen Hospital, Capital Medical University (2018-YJJ-zzL016).

## Conflict of Interest

The authors declare that the research was conducted in the absence of any commercial or financial relationships that could be construed as a potential conflict of interest.

## Publisher's Note

All claims expressed in this article are solely those of the authors and do not necessarily represent those of their affiliated organizations, or those of the publisher, the editors and the reviewers. Any product that may be evaluated in this article, or claim that may be made by its manufacturer, is not guaranteed or endorsed by the publisher.
